# Reply to Santini et al.: Total population reports are necessary for global biomass estimation of wild mammals

**DOI:** 10.1073/pnas.2316314121

**Published:** 2024-01-16

**Authors:** Lior Greenspoon, Yuval Rosenberg, Shai Meiri, Uri Roll, Elad Noor, Ron Milo

**Affiliations:** ^a^Department of Plant and Environmental Sciences, Weizmann Institute of Science, 76100 Rehovot, Israel; ^b^School of Zoology, Tel-Aviv University, Tel-Aviv 6997801, Israel; ^c^The Steinhardt Museum of Natural History, Tel Aviv University, Tel-Aviv 6997801, Israel; ^d^Mitrani Department of Desert Ecology, The Jacob Blaustein Institutes for Desert Research, Ben-Gurion University of the Negev, Midreshet Ben-Gurion, 8499000, Israel

**Fig. 1.**
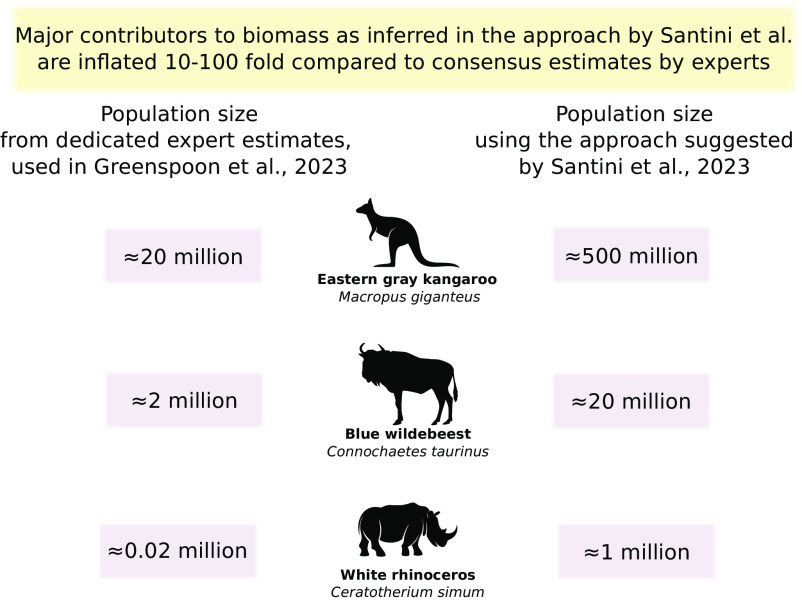
We replicated the analysis presented in Fig. 1*B* by Santini et al. They inferred the total biomass of 158 species from local population density measurements, arriving at 55 Mt. However, ≈85% of said biomass is concentrated in 10 species, three of which are in the example above. These 10 species are all large-bodied, highly monitored, and surveyed over large areas to produce expert-based abundance assessments (the full table can be found in (3)). It is unreasonable to assume that local densities extrapolated naively are more reliable than expert assessments. For example, using the method suggested by Santini et al, the estimated number means that Eastern gray kangaroos outnumber humans in Australia 20 to 1.

A recent comment by Santini et al. (1) refers to our (2) efforts to quantify mammal biomass globally. However, we think that they misunderstood our paper's main aim. They suggest an alternative method which, as we show below and in Fig. 1, gives unreasonably high global population size estimates compared to the scientific consensus. Their comment also reflects several points of miscommunication regarding our methodology, which we address in Table 1 and in a point-by-point document (3).

**Table 1.** List of main claims by Santini et al. and clarification of the misunderstanding or remaining disagreement

Claim by Santini et al.	Clarification
“We could not replicate the results of 7 of the top 10 marine mammals' biomass due to inconsistent use of RL data”	While rechecking values following this correspondence we found a few minor errors in the total population within those species. The total effect of all corrections on marine mammal biomass is negligible (<1%). We updated the values in our original paper's gitlab repository. We note that extracting data manually from heterogenous texts for hundreds of mammal species is a task that can contain some misinterpretations and manual errors.
“Under-estimation of uncertainty”	Saitini et al. seem to have misinterpreted the conservative approach we took which aimed to avoid underestimation of uncertainty. We analyzed and collected uncertainty reports for species with the largest mass contribution for which confidence intervals were available and found that all of them were smaller than twofold. To be conservative, for each of the species with reported population abundances, we set 95% CI of twofold above and below the estimated values.
“Sum of inconsistently estimated population sizes.” Examples given: White-tailed deer (*Odocoileus virginianus*)	We used the best available sources of data and documented their use in detail. It seems highly unlikely to us that a dedicated analysis on the white-tailed deer's abundance by experts from the US wildlife state agencies across its American range (which we have used) is less accurate than the simple extrapolation suggested based on few local densities that may not be representative of the huge range of habitats and climatic conditions inhabited by this species.
“Inconsistent use of RL reported populations, sometimes taken from the description, sometimes from the reported total, sometimes neither.” Examples given: Malagasy giant rat (*Hypogeomys antimena*)	Total population was taken from RedList population abundance estimates, either from the reported total or the description sections of theRedList information sheets as this value does not always appear in the same textual location. Regarding the specific example of *H. antimena* population, our data were curated in late 2019 as detailed in the paper, while the abundance has since been updated (2022) by RedList experts. The original values extracted can be found in our gitlab repository. The species’ total contribution to the total biomass of wild land mammals is ≈50 tons out of the estimated ≈20 million tons. Following the suggested update, the contribution of the species would be on the order of only ≈5 tons. In both cases, the contribution is negligible (<0.001%).

Due to format limitations, we address all of the claims by Santini et al., in a point-by-point document (3).

We reiterate that we did not intend or claim to use population-size data to infer field densities of specific mammal species nor did we report calculated densities. Our goal was to estimate the combined global biomass of all ≈5,000 mammal species.

Our analyses used RedList global abundance estimates, which has limitations and biases but is the only systematic global dataset of its kind. Santini et al. suggest instead to simply multiply locally measured densities from their published dataset (4) with range size to achieve global abundance estimates. While working on our published manuscript, we considered this approach and discussed it with Prof. Santini but found that it yields unrealistic results. For example, applying their approach for the blue wildebeest, one of the world’s best-monitored species, yields a global abundance estimate 10 times higher than the undisputed global values provided by experts over many years. The approach by Santini et al. provides similarly unrealistic results for many highly monitored species, as we show in Fig. 1.

Population densities vary dramatically across a species’ range, and researchers mostly study animal populations where they are abundant (5). Consequently, locally measured population densities tend to be biased toward maximal densities (5) and are not representative of species’ mean densities across their entire extent of suitable habitat (6, 7). Additionally, the Santini et al. 2022 dataset contains measurements from 1970 to 2020, during which many populations suffered sharp declines (8)—another plausible cause of over-estimation. Moreover, using locally measured densities to extrapolate abundances over large areas, which may include distinct habitats, can lead to unrealistic global abundances (9; Fig. 1 and Table 1).

Many large-bodied mammal species’ total population sizes [which contribute most to the global wild mammal biomass (2)] are assessed using multiple methods over long time periods. It is unlikely that experts are underestimating global populations of species such as the wildebeest, white rhinoceros, and others by several orders of magnitude, as suggested by Santini et al. (Fig. 1). Consequently, we think that our chosen approach to estimate global mammals’ biomass is sound given current data.

Finally, our estimates may contain large uncertainties at the species level. However, the total relative uncertainty is substantially reduced by summing biomass across many species, as we do. Better and more standardized measurement of animal populations will certainly improve the census of animal biomass; we hope our paper motivates the scientific community to focus more resources on this urgent effort.
